# Evaluation of ultrasound velocity in enucleated equine aqueous humor, lens and vitreous body

**DOI:** 10.1186/s12917-014-0250-3

**Published:** 2014-10-14

**Authors:** Ulrike Meister, Bernhard Ohnesorge, Daniel Körner, Michael H Boevé

**Affiliations:** Stiftung Tierärztliche Hochschule Hannover, Klinik für Pferde, Bünteweg 9, 30559 Hannover, Germany; Theoretisch-Physikalisches Institut, Friedrich-Schiller-Universität Jena, 07743 Jena, Germany

**Keywords:** Equine eye, Ultrasound velocity, Intraocular lens, Aqueous humor, Vitreous body

## Abstract

**Background:**

Sonographic ophthalmic examinations have become increasingly important in veterinary medicine. If the velocity of ultrasound in ocular tissues is known, the A-mode ultrasound method may be used to determine the axial intraocular distances, such as anterior chamber depth, lens thickness, axial length of the vitreous and axial globe length, which are required for intraocular lens (IOL) power calculations. To the authors' knowledge, the velocity of ultrasound in the ocular tissues of the horse was not previously determined. In the present study, 33 lenses, 29 samples of aqueous and 31 of vitreous from 35 healthy equine eyes have been examined. The corresponding ultrasound velocities are reported in dependence of age, temperature, gender and elapsed time after enucleation.

**Results:**

The velocity of ultrasound at 36°C in equine aqueous, lens and vitreous are 1529 ±10 m/s, 1654± 29 m/s and 1527 ±16 m/s respectively, and the corresponding conversion factors are 0.998± 0.007, 1.008 ±0.018 and 0.997 ±0.010. A linear increase of the speed of ultrasound with increasing temperature has been determined for aqueous and vitreous. No temperature dependence was found for the speed of ultrasound in the lens. The ultrasound velocity did not significantly differ (95%) on the basis of gender, age or time after enucleation during the first 72 hours after death.

**Conclusions:**

Compared to human eyes, the ultrasound velocity in equine lental tissue deviates by one percent. Therefore, axial length measurements obtained with ultrasound velocities for the human eye must be corrected using conversion factors. For the aqueous and vitreous, deviations are below one percent and can be neglected in clinical settings.

## Background

In addition to a full ophthalmological examination, evaluation of the globe can be achieved by ultrasonography, which is used routinely in human medicine and has become increasingly important in veterinary medicine. Ultrasonographic examination is particularly important in the case of opacifications of the ocular media, e.g. by cataract. In veterinary ophthalmology, B-mode (brightness modulation) ultrasonography is used regularly. It provides a two-dimensional image that resembles a cross-section of the examined tissue. A-mode (amplitude modulation) ultrasound, e.g. for biometric measurements, is less commonly used. In human ophthalmology, the A-mode ultrasound is used before cataract surgery to determine the required dioptic power of the artificial intraocular lens (IOL) and to monitor the postoperative state of the eye [1].

Accurate knowledge of the axial intraocular distances such as anterior chamber depth, lens thickness, axial length of the vitreous and axial globe length is necessary for IOL power calculations and the transversal size of the lens is important for the choice of the proper IOL size [2-4]. The method of choice is the biometric A-scan [5] which has previously been described for dogs [6], cats [7] and horses [8]. The intraocular distances (*l*) are calculated by determination of the travel time (*t*) of an ultrasonic wave with velocity (*c*) according to the formula *l*=*ct*. The majority of biometric studies of animal eyes are performed with an ultrasound machine that has been calibrated for humans. The travel time measurement is converted internally to a length measurement *l* using the known ultrasound velocity *c*_*h*_ for the human eye. However, the ultrasound velocity differs among species and the true length *L* is given by *L* = *f* ⋅ *l* and *f* = *c*_*s*_/*c*_*h*_, the conversion factor, where *c*_*s*_ is the ultrasound velocity of the examined species. It is therefore necessary to know the exact velocity of ultrasound propagation in the different ocular media for the species examined. In general, the ultrasound velocity depends upon different factors, including the ultrasound frequency and the type and temperature of the tissue examined.

The ultrasound velocity through ocular tissues has already been examined for humans and several animal species [2,9-15]. These studies indicate that the values for the velocity of ultrasound in lental tissue differ considerably among species, whereas no difference for the aqueous and vitreous is reported. The axial lens thickness is an important factor for the calculation of dioptic IOL power following the usual formulas of Retzlaff and Binkhorst and its accurate determination is, hence, very important [16]. A deviation of 50 m/s for the speed of ultrasound in a human lens already leads to uncertainty potential aberration of 0.5 D for the IOL power [17]. Therefore, considering different species, it is generally insufficient to estimate the IOL power by simply using the ultrasound velocity of the human lental tissue. The exact velocity for the horse should be used to achieve emmetropia in the eye. Visual soundness is very important for the pleasure and performance horse. A visually compromised horse may show abnormal behaviour and the rider faces an unpredictable, frightened animal [18].

To the authors’ knowledge, the velocity of ultrasound in the ocular tissue of the horse has not been examined previously. The purpose of this study was to evaluate the velocity of ultrasound in equine aqueous humor, lens and vitreous in relationship to the factors age, temperature, gender and elapsed time after enucleation. The corresponding conversion factors have been calculated and implications for clinical ophthalmology are discussed.

## Methods

Equine eyes were obtained from several slaughterhouses. Post mortem, every globe was enucleated and macroscopically examined. Afterwards the globes were immediately placed in a saline solution (0.9% Sodium chloride). All samples were cooled during transport. Only eyes without any signs of previous ophthalmic disease such as cataract or liquefaction of the vitreous body were examined. Depending on storage and the elapsed time after enucleation, the eyes were divided in three groups. Group A consists of 12 lenses, 10 samples of aqueous and 10 of vitreous, and the ocular tissues were stored at 8°C and examined within 12 hours after death. The eyes in group B were stored at 4°C and examined within 48 hours after death. This group consists of 11 lenses, 13 samples of aqueous and 12 of vitreous. Group C consists of eyes that were frozen at −18°C and examined within 72 hours after death, which amounts to 10 lenses, 6 samples of aqueous and 9 of vitreous. For some eyes, it was not possible to harvest enough aqueous or vitreous tissue for measurements. In addition, two lenses were damaged during the dissection process and excluded from the study.

Prior to examination, all eyes were adjusted to room temperature. In total, 35 healthy eyes were used for this study, out of which 17 eyes were from female and 18 from male horses. The age of the horses ranges from 6 to 22 years.

Preparation of the enucleated eyes started for group A within 12 hours, for group B within 48 hours and for group C within 72 hours post mortem by obtaining samples of the aqueous and vitreous, which were then stored in the sample-holders. The sclera was removed using scissors and the cornea was carefully separated to preserve the lens. Any remaining zonular material and vitreous was removed and the lens was placed in its respective sample holder. The examination of all ocular tissues occurred using three specially crafted sample holders, which consist of a copper frame to enable quick thermalisation and two Plexiglas plates, one of which was removable. The tissue sample was placed between the Plexiglas plates. The sample holder for the lens, aqueous and vitreous had sizes of 10 mm, 7 mm and 5 mm, respectively, in order to enable the tissue to be in full contact with the Plexiglas plates, thus securing a connection of the ultrasound signal between sample and holder without trapped air. By using sample holders with a fixed extent, the thickness of the tissue to be examined was determined accurately. In particular, measurement errors of the ultrasound velocity in the lens due to deformation for the length measurement, e.g. by the use of a manual calliper, could thus be avoided. The travel time of the ultrasonic wave was measured using the impulse-echo method, which requires only a single transducer for ultrasound emission and registration. The ultrasound probe was directly connected to the first Plexiglas plate of the sample holder and a part of the ultrasound signal was transmitted through the sample (A-scan). Due to the difference of the refractive index between the Plexiglas and the sample, a clear signal could be assigned to the reflected wave corresponding to the transition between the first Plexiglas plate and the sample, as well as for the transition from the sample to the second Plexiglas plate. Therefore, a direct determination of the travel time (*t*) of the ultrasonic wave through the sample of thickness (*d*) was possible. Different setups for the measurement of the velocity of ultrasound such as the substitution technique or the double transmission technique have been described previously [9,10,19,20]. In the present study the double transmission technique was used and the velocity (*c*) is then provided by the formula *c*=2*d*/*t*.

The speed of ultrasound depends on the frequency of the ultrasound waves. For the present study, ultrasound equipment with a frequency range of 1 to 4 MHz was available. A 4 MHz transducer was used throughout. In order to obtain reference values, the ultrasound velocity for distilled water was measured for each sample holder separately and used to calibrate the experimental setup.

Temperature-dependent changes in the velocity of ultrasound were determined from 32°C to 40°C in 2°C increments by placing the sample holders in a temperature-controlled water bath. For each measurement, five minutes of temperature adjustment were given.

For the velocities and conversion factors, the average and the standard deviation from the respective data subsets have been calculated. All hypotheses are evaluated using an appropriate variant of Student's *t*-test using a significance level of *α*=0.05. Regression curves are computed using the method of least squares and for linear regressions we give the value of Pearson's correlation coefficient *r*, which is related to the coefficient of determination *R*^2^ by *R*^2^=*r*^2^.

## Results

**Figure 1:**
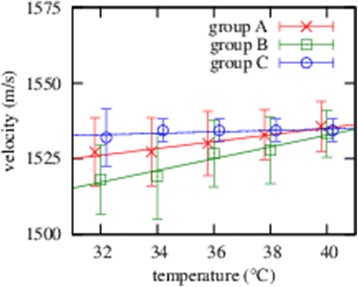
**Ultrasound velocity in the equine aqueous for three separate sample groups is depicted depending on the temperature.**

**Figure 2:**
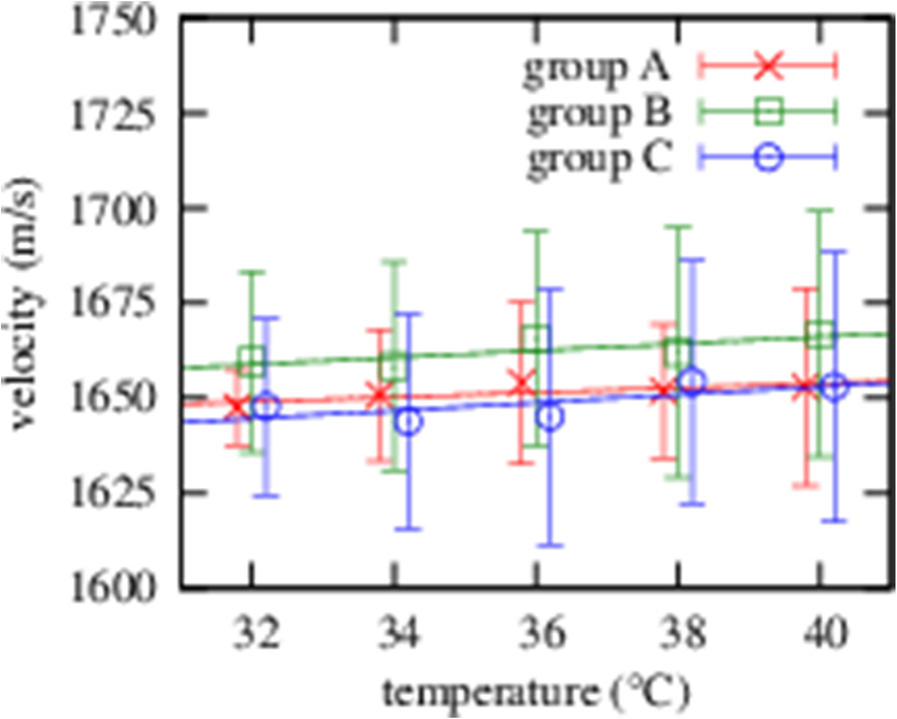
**Ultrasound velocity in the equine lens; The ultrasound velocity in the equine lens is depicted depending on the temperature for three separate sample groups at different elapsed times after enucleation and storage.**

**Figure 3:**
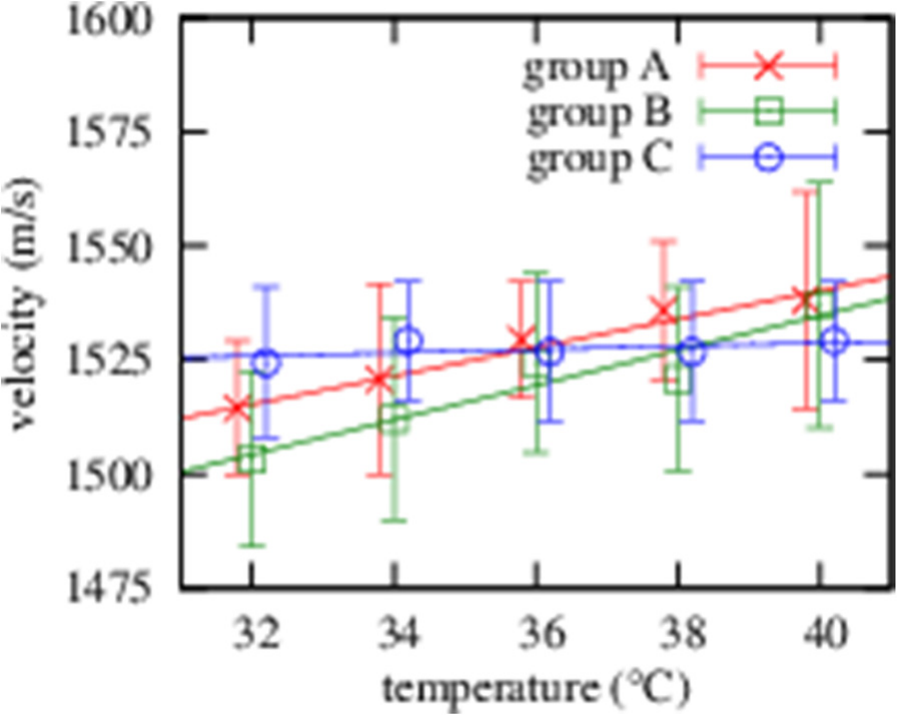
**Ultrasound velocity in the equine vitreous is depicted depending on the temperature for three separate sample groups is shown.**

Figures 1, 2 and 3 show the ultrasound velocity in the equine aqueous, lens and vitreous depending on the temperature for three separate sample groups that differ in time after enucleation and storage. The mean velocity for each tissue did not significantly depend (95%) on the time after enucleation and storage.

Hence, values for all sample groups have been averaged and the values for the velocity of ultrasound through aqueous, lens and vitreous at 36°C were 1529 ± 10 m/s, 1654 ± 29 m/s and 1527 ± 16 m/s and the conversion factors were 0.998 ± 0.007, 1.008 ± 0.018 and 0.997 ± 0.010.

**Figure 4:**
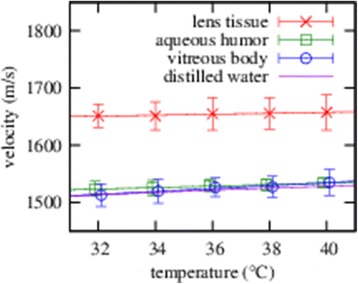
**Ultrasound velocity in the equine aqueous, vitreous and lens tissue is depicted, averaged over all three sample groups, depending on the temperature of the sample.**

**Figure 5:**
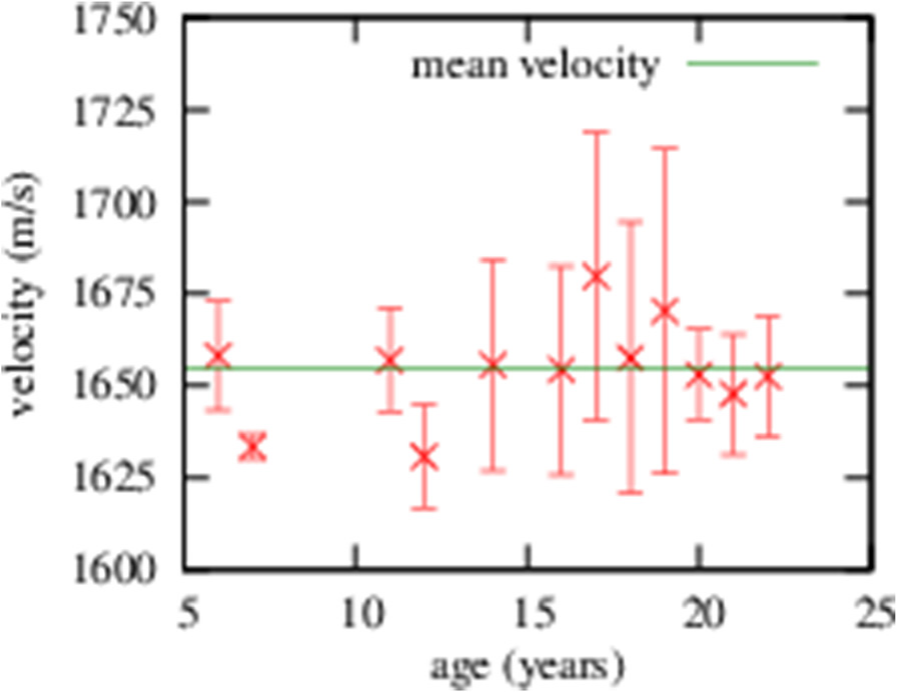
**The ultrasound velocity in the equine lens tissue is averaged over all sample groups and temperatures and depicted depending on the age of the respective horse.**

A linear regression curve was plotted for the data averaged over all three sample groups. The result of this is shown in Figure 4 and the value of the linear coefficient for lental tissue, which characterises the temperature dependence, is not significantly different from zero (95%). In contrast, the velocity of ultrasound in aqueous and vitreous increases significantly (95%) with increasing temperature and this was confirmed by a linear correlation. The corresponding regression formulas are depicted in Figure 4 and amounted to *c*(*m*/*s*) = (1.23 ± 0.13) ⋅ *T*(*°C*) + (1485 ± 5) for the aqueous with correlation coefficient *r*=0.98 and *c*(*m*/*s*) = (2.63 ± 0.33) ⋅ *T*(*°C*) + (1430 ± 12) for the vitreous with correlation coefficient *r*=0.98. No significant difference (95%) in the velocity of ultrasound between male and female individuals could be demonstrated. The temperature-averaged ultrasound velocity through the lens for horses of different ages is shown in Figure 5. For each data point, the deviation from the mean velocity is not significant (95%) and this result is consistently reflected by the low correlation coefficient *r*=0.36 for a simple linear correlation. Furthermore, we found that the ultrasound velocities through the aqueous and vitreous do not significantly depend (95%) on the age of the horse.

## Discussion

We have calculated conversion factors for the three types of healthy equine ocular media, which are useful to correct the calculations of axial intraocular distances that are based on the speed of ultrasound in the human eye. The speed of ultrasound in human aqueous, lens and vitreous at 37°C are 1532 m/s, 1641 m/s and 1532 m/s respectively [21]. The corrections for aqueous and vitreous are on a sub-percent magnitude and it is therefore safe to neglect these in a clinical setting. The correction for the equine lens, which is 1.008(18), is on a percent-level and in this respect closer to the human lens than to the values that have been reported for pig, dog and rabbit lenses, which are 1.024, 1.040 and 1.055 respectively [10].

The present study shows that the speed of ultrasound through the optical media of the equine eye does not change during the first 72 hours after enucleation. In a previous study in human eyes it was concluded that there is no measurable change in the velocity of ultrasound in ocular media during the first few days after death [9]. Another study concerning the structure and regional water content of lenses in humans, cattle and pigs concluded that there are no changes in structure and water mobility within the first 3 days after death [22]. Also the storage did not influence the speed of ultrasound. This was proved by our statistical analysis. As well in a previous study, which analyzed the thermal stability characteristics of the lenses of vertebrates, no effects of freezing on stability properties of the lens were observed [23]. We therefore assume that our results closely resemble the ocular ultrasound velocities in the living animal.

For the present study a 4 MHz transducer was used throughout, which is not the commonly used frequency for ocular ultrasound measurements. Axial length measurements with A-mode are generally conducted with a frequency of 8 MHz and B-mode ultrasonography with a frequency of 10 MHz [3]. In previous studies a range of different ultrasound equipment has been used, operating at frequencies of 4 MHz [9], 10 MHz [2,10,19] or 20 MHz [24]. The differences in the ultrasound velocity for separate frequencies have been described to be less than one percent [25]. We are therefore confident that it is sufficient to use a single frequency for all measurements.

Previous studies have shown that the human central corneal surface temperature is 34.8°C, which is 2.2°C lower than the average physiological body temperature [26]. Other studies have indicated that the temperature within the globe increases from the cornea towards the more posterior structures [26]. The anterior lens surface temperature in rabbits has been shown to be approximately 36°C, whereas the posterior lens surface was 38°C [10]. Since the physiological body temperature of the horse varies between 37.5°C and 38.2°C, we assume the equine corneal temperature to be in the range of 35.3°C to 36.0°C.

As visible in Table 1 the velocity of ultrasound in equine aqueous and vitreous matches values for a broad range of different species like human, bovine, canine, rabbit, porcine and camel [9,10,13,21,27]. Both values lie slightly above the ultrasound velocity of distilled water, which is 1521 m/s at 36°C, and this further supports the hypothesis that the speed of ultrasound in aqueous and vitreous is dominated by its water content for all species. Consequently, we found that the speed of ultrasound in these ocular tissues increases with increasing temperature, which has been described for other species as well [2,10]. In contrast, our results for the velocity of ultrasound in equine lenses do not indicate a temperature dependence. However, another study reports an increase of the speed of ultrasound in porcine ocular lental tissue with rising temperature [12]. The considered temperature range in this study is greater (23°C – 37°C) than in the present study. Yet, the regression formula c =1642,61 +1,00 x T (m/s) shows that the temperature dependence is very small. It is known that the speed of ultrasound of a tissue highly depends on its content of protein and water. The temperature dependence of ultrasound is mainly determined by the respective dependence of the compressibility. For water, the compressibility decreases for increasing temperature up to a minimum at 67°C, i.e. the ultrasound velocity increases. For all other liquids however, the ultrasound velocity decreases and the sign of the temperature coefficient is different. The human lens consists of about 66% of water and 34% of protein [22]. Concerning lental proteins, two groups can be distinguished, soluble and insoluble, and the ratio of these vary with species and age [3]. The water content of the lens, and thus the velocity of ultrasound (Table 1), also varies among species [22] and lenses are therefore expected to show a different behaviour with changes of temperature.

**Table 1:** **Compilation of results from the literature for the ultrasound velocity in different ocular tissues**

**Study**	**Species**	**Temp.**	**c aqueous (m/s)**	**c vitreous (m/s)**	**c lens (m/s)**
Jansson and Kock [9]	Man	37°C		1532 ±0.5	1641 ±1.2
Oksala & Lehtinen [13]	Cattle	22°C	1495	1495	1650
Coleman, Lizzi [21]	Man		1532	1532	1641
Görig [10]	Dog	36°C		1532 ±0.7	1707 ±19
Görig [10]	Rabbit	36°C		1531 ±1.1	1731 ±21
Görig [10]	Pig	36°C		1532 ±0.8	1681 ±6.3
Osuobeni and Hamidzada [11]	Camel	20°C			1686 ±16
Hamidzada and Osuobeni [27]	Camel	20°C	1499 ±23	1497 ±24	
Present study	Horse	36°C	1529 ±10	1527 ±16	1654 ±29
Coleman, Lizzi [20]	Man Cataract	37°C			1629 ±38

In general, the amount of water in the lens consists of free and bound water. The ultrasound velocity of free water is lower than of bound water. The cortex has the highest percentage of free water, whereas the nucleus consists of a low amount of free water and a high amount of protein-bound water [22]. Hence, it can be expected that the speed of ultrasound in the lental periphery (cortex) is slower than in the nucleus [24]. The equipment used in this study was not suited to resolve these differences and we determined the ultrasound velocities in average for the lens as a whole.

The amount of free water in the human lental nucleus increases during the aging process [22,28] and a decrease of the ultrasound velocity for older horses may therefore be expected. However, in the present study, no significant correlation (95%) between age and velocity of ultrasound for horses between 6 and 22 years of age could be determined. Due to a lack of samples, no conclusion can be drawn for horses older than 22 years. In a study of human lenses with a wider age distribution, a correlation between the velocity of ultrasound and age was found [20].

In a previous study it was reported that the speed of ultrasound is slower in cataractous lenses, compared to healthy lenses, because of the increased water content [20]. Another study describes that during cataract formation the content of bound water in the lental nucleus is decreasing in favour of “free” water [22]. Therefore, the ultrasound velocity decreases as well. For a correct determination of the axial ocular dimension in equine cataractous eyes further evaluations of such eyes are necessary.

## Conclusions

The main result of this study is the calculation of the conversion factors which are essential to correct axial ocular dimensions measured with ultrasound equipment calibrated for human use. We found that the conversion factors for the aqueous and vitreous can safely be ignored. However, for the equine lens, the ultrasound velocity is significantly higher (95%) than in the human lens and measurements must be corrected.

In the range from 32°C to 40°C no significant temperature dependence (95%) was found and our estimate for the conversion factor of 1.008 for the lens may therefore be used throughout.

The IOL power calculation is particularly important for cataract surgery. Following the usual formulas of Retzlaff and Binkhorst, essential factors include the curvature of the cornea, the axial length and the postoperative anterior chamber depth, which in the equine eye consists of the anterior chamber depth plus ½ lens thickness [16]. Variations of these values results in dramatically different dioptic power of the IOL implant to be inserted [29]. However, judging from the results of a study [20] relating to human cataractous lenses, we hypothesise that the ultrasound velocity in the cataractous equine lens is substantially lower than for the healthy lens. Usage of the conversion factor for the healthy lens will result in an overestimation of the IOL power. We therefore stress that further studies in cataractous lenses are needed to allow for exact results in those cases.

## Abbreviations

IOL: Intraocular lens
D: Dioptre
m/s: Meter per second
A-mode: Amplitude modulation
B-mode: Brightness modulation
°C: Centigrade
MHz: Megahertz
mm: Millimeter
